# Draft Genome Sequence of Pseudomonas fluorescens SRM1, an Isolate from Spoiled Raw Milk

**DOI:** 10.1128/genomeA.00138-15

**Published:** 2015-03-19

**Authors:** Raquel Lo, Mitchell J. Stanton-Cook, Scott A. Beatson, Mark S. Turner, Nidhi Bansal

**Affiliations:** a School of Agriculture and Food Sciences, The University of Queensland, Brisbane, Queensland, Australia; b School of Chemistry and Molecular Biosciences, The University of Queensland, Brisbane, Queensland, Australia; c Queensland Alliance for Agriculture and Food Innovation, The University of Queensland, Brisbane, Queensland, Australia

## Abstract

Pseudomonas fluorescens is considered a major milk spoilage organism due to its psychrotrophic nature and ability to produce heat-stable proteases and lipases. Here, we report the draft genome and annotation of P. fluorescens SRM1 isolated from spoiled raw milk and the presence of an operon encoding spoilage enzymes.

## GENOME ANNOUNCEMENT

Pseudomonas spp. are ubiquitous environmental bacteria commonly found in soil and water and on the surface of fruits and vegetables (1) and are the major pathogens that spoil foods stored aerobically under refrigerated conditions (2, 3). Milk is commonly spoiled by Pseudomonas spp. via postpasteurization contamination or by the production of heat-stable lipases and proteases before pasteurization (4). Pseudomonas-derived proteases and lipases can lead to degradation of milk protein and fat, resulting in bitterness, gelation, and rancidity (5–7). The most common species of Pseudomonas that causes milk spoilage is Pseudomonas fluorescens (8–10). As of January 2015, 5 complete and 38 draft genome sequences of P. fluorescens are available; however, none of these strains were isolated from milk. Here, we present the draft genome sequence of Pseudomonas fluorescens SRM1, a psychrotrophic isolate obtained from spoiled raw milk stored at 4°C.

Genomic DNA was submitted to Macrogen (Seoul, South Korea) for sequencing with Illumina HiSeq2000 (San Diego, CA, USA). A total of 9,890,048 paired-end reads, each 101 bp long, were obtained. The reads were quality trimmed by Nesoni clip (https://github.com/Victorian-Bioinformatics-Consortium/nesoni) and assembled *de novo* by Velvet (11) with a k-mer of 67, resulting in 409 contigs and 78 scaffolds. The *N*_50_ of the scaffolds was 162,120-bp, and sequence coverage was approximately 113-fold. Using Mauve Contig Mover (12), the scaffolds were ordered against the genome of P. fluorescens Pf0-1, a soil isolate (13), which was found to be the closest match among fully sequenced P. fluorescens by Kraken (14). The assembled genome has a size of 6,352,984 bp and G+C content of 59.4%. Annotation by PROKKA (15) predicted 5,829 protein-coding sequences.

P. fluorescens SRM1 was able to coagulate milk when inoculated as a pure culture (current study), which agrees with the presence of the thermostable extracellular protease encoding gene, *aprX* (alkaline protease) (16). This gene is the first in an operon containing genes encoding a protease inhibitor (*inh*), an ABC transporter (*aprDEF*), two serine proteases (*prtA* and *prtB*), and two lipases (*lipA* and *lipB*). This operon has been identified in the raw milk isolate P. fluorescens B52, which contains only one lipase gene (17), and part of this operon was also found in several strains of dairy and non-dairy origin (16, 18–20). The ABC transporter directs the secretion of the protease and lipase(s) (18, 21), while the serine proteases are predicted to be autotransporters. This operon is also conserved in the five fully sequenced P. fluorescens strains, except that only P. fluorescens Pf0-1 has two lipase genes and two other strains lack the serine protease genes. Compared to the fully sequenced strains and strains in which this operon was characterized, the genomic context and the encoded enzyme sequences are most similar to those of the soil isolate Pf0-1. The publication of this genome sequence will allow comparison between food and environmental isolates of P. fluorescens and assist the study of food spoilage bacteria.

### Nucleotide sequence accession numbers.

The P. fluorescens SRM1 genome sequence and annotation data have been deposited in the European Nucleotide Archive under the accession number CDMF01000001. The version described in this paper is the first version, CDMF01000001.1.
